# Drivers of consumer food choices of multinational corporations’ products over local foods in Ghana: a maximum difference scaling study

**DOI:** 10.1186/s12992-024-01027-x

**Published:** 2024-03-19

**Authors:** Eric Nyarko, Tina Bartelmeß

**Affiliations:** 1 Department of Statistics and Actuarial Science, School of Physical and Mathematical Sciences, University of Ghana, Box LG 115, Accra, Legon Ghana; 2 Faculty of Life Sciences: Food, Nutrition and Health, University of Bayreuth, Fritz-Hornschuch-Strasse 13, Kulmbach, Germany

**Keywords:** Nutrition transition, Multinational food corporations, Supermarkets, Fast-food, Consumer preferences, Ghana

## Abstract

**Introduction:**

The fundamental transformation of food systems and retail environments in low-income countries is influencing consumers' food choices and dietary habits in unfavourable directions through the consumption of highly processed, energy-dense foods, predominantly manufactured by multinational food corporations. This study aims to identify the principal factors driving consumers' preference for multinational foods over local foods in the urban Accra region of Ghana.

**Method:**

This cross-sectional survey involving a random sample of 200 consumers conducted in March/April 2023 using interviewer-administered questionnaires employed a maximum difference scaling approach to investigate the drivers of urban Ghanaian consumer food choices for multinational food corporations' products over local foods. The maximum difference scaling modelling analysis utilized in this study identifies the primary drivers of multinational food corporations' product preferences and the associated trade-offs.

**Result:**

The study discovered that food quality and safe packaging, perceived healthiness, taste and flavour, and nutritional value were the most significant factors driving consumer preference for multinational food corporations' products over local foods in Ghana. The criterion food quality and safe packaging had the significantly highest utility than all other attributes in terms of consumer preference for products/meals from multinational food corporations over local foods.

**Conclusion:**

The results of this study provide significant contributions to the existing body of research, as previous studies have not identified these factors as primary drivers of multinational food products. Public health authorities and nutritionists can use the study's findings to implement targeted quality assurance measures in local markets and to address the drivers in health education campaigns.

## Introduction

The impact of multinational food corporations in emerging economies on the nutrition transition [1, 2], has been a long-standing public health concern [3]. Multinational food corporations such as fast-food restaurants, manufacturing and processing corporations and retailers increasingly dominate global trade and investment and are progressively penetrating markets in low-income countries [4, 5]. Various supply chain processes facilitate the expansion of multinational corporations to low-income countries, including trade liberalization, market concentration in the food system, and foreign direct investment (FDI) [6–8]. FDI serves as a mechanism through which corporations can enter the markets of Global South countries and acts as a stimulus for the globalization of the highly processed food industry and the economic development of a country [6]. However, FDIs predominantly occur in the context of food processing and enable the globalization of the highly processed food industry and the production and distribution of such foods in these markets, which pose significant challenges to public health nutrition [1, 6, 9]. It is argued that this profound change in food systems in low-income countries is influencing consumers’ food choices and dietary habits in an undesirable direction of consuming highly processed, energy-dense foods [1, 5, 10]. These developments are accelerating the so-called nutrition transition [1], which leads to an increase in the double burden of malnutrition, a growing public health problem in sub-Saharan African countries [9].

Considerable research efforts have been dedicated to elucidating the unfavourable health outcomes linked to the increasing availability and accessibility of highly processed food and beverages in low-income countries [2]. The causal association between the consumption of highly processed foods and non-communicable diseases (NCDs), such as type 2 diabetes, obesity, and coronary heart disease, has been well-established. Unhealthy dietary habits are a significant global risk factor for NCDs, and enhanced public health nutrition measures can aid in preventing and addressing unhealthy dietary habits in populations [11, 12]. Consequently, improving population nutrition and food environments has emerged as a crucial public health priority [13]. In addition to changing conditions, public health approaches have largely centred on altering individual behaviours [14]. Sophisticated research is needed to identify the drivers of consumer choice for global food products in emerging economies and to examine how they interact with changing food environments [12, 15].

To date, scholarly research has given relatively less attention to examining the perspective of consumers and demand-side factors in low-income countries regarding global food products [15, 16]. Previous research has primarily focused on the supply-side processes and their connections to the nutrition transition [17]. Scholars have tacitly assumed that the proliferation of multinational food corporations in emerging economies, along with associated marketing efforts, not only alters the food environment and the availability of food products but also shapes food preferences and fosters the appeal of global food products [17, 18]. The spread of global food products in emerging economies is presumed to result in a gradual change in local food culture, by transferring tastes, preferences, and habits from highly developed countries to low-income countries, particularly through advertising and promotion [17, 19]. Nonetheless, the socio-cultural and nutritional contexts of a country, as well as the socio-economic characteristics of consumers, influence the degree of acceptance of global food products and the motives underlying consumer choices. Prior research on the consumer acceptance of multinational corporations' food products in low-income countries has predominantly suggested that these products are valued for their symbolic and status-enhancing properties, as well as their perceived foreignness or non-localness, which are considered prestigious and cosmopolitan, implying a social signalling effect [18]. However, at the time of these studies, research on nutrition and consumption patterns in low-income countries was primarily concerned not only with the malnutrition of low-income groups, but also with the slightly aspirational middle class, with their purchasing power and their purported aspiration for a Western lifestyle [2]. In the meantime, the middle class in Sub-Saharan African countries has gradually increased, and due to economic growth, improved living standards, urbanization, and progressive cultural globalization [12], it can be assumed that, in addition to the global appeal of multinational corporations' food products, other drivers for their consumption have become increasingly important.

This study investigates the drivers of urban Ghanaian consumer food choices of multinational food corporations' products over local foods using a maximum difference scaling approach to identify current key drivers. The study's results can be used to formulate recommendations for public health nutrition policy makers to support healthy food choices in Ghana.

## Impact of multinational food corporations on consumer food choices in Ghana

### Multinational food corporations and health impacts on Ghanaian consumers

At the global level, changes in the supply chain control have led to the concentration of multinational food and beverage manufacturers, retailers, and fast-food takeaways in the food sector, which has increased the availability of highly processed, packaged, and unhealthy foods and beverages. This phenomenon has been linked to the rising prevalence of overweight and obesity, particularly in urban areas, of emerging economies [20, 21]. In Ghana, a prominent emerging economy in sub-Saharan Africa, foreign direct investment in highly processed foods has primarily led to breweries and distilleries, sugar and confectionery as well as soft drinks being more widely available. In addition, the retail sector and the density of franchise fast food restaurants have also increased [22]. No specific data is available for Ghana, but an overall comparison of African regions shows that most FDI flows have been to West Africa, partly because Guinness Ghana owned by Diageo invested substantially in Ghana in the early 2000s [23]. In Ghana there has been a correlation between economic growth and a decrease in undernutrition over the past decade, although significant disparities persist between rural and urban populations and between the northern and southern regions [12]. However, in the southern region and urban areas in particular, the escalating issue of health risks associated with the rise of overweight and obesity has become a cause for concern [15]. According to the Global Nutrition Report [24], the prevalence of overweight in Ghana among women and men in 2019 was 43.3% and 23.9%, respectively, and the prevalence of obesity was 19.3% and 5.6%, respectively.

The Ghanaian food environment is gradually changing, with multinational food corporations offering an increasing number of products and meals, especially in urban areas [12, 21]. In Ghana, prominent multinational corporations in the food processing and beverage sector include Nestlé, Danone, Guinness Breweries and Coca Cola Bottling Company. Major supermarket chains operating in the country include Shoprite Holdings, Melcom Group, PICK 'N PAY, and SPAR. Fast food companies with branches in Ghana include Kentucky Fried Chicken, Burger King, and Pizza Hut. As a result of the presence of multinational corporations in the Ghanaian market, the consumption of traditional foods is reportedly declining [25], as has been observed in other Global South countries [10]. The shift from urban markets selling fresh produce to commercially prepared and processed foods is seemingly contributing to the trend towards processed and imported foods in urban diets, as it is assumed that dietary preferences are changing and access to nutrient-rich foods is restricted by the built environment [12, 15]. Studies in Sub-Saharan Africa including Ghana show that the rapid spread of supermarkets has fundamentally altered the local food retail environment and has negatively affected customers' nutritional outcomes [26–28]. Survey and panel data analyses reveal that customers who shop at supermarkets are more likely to consume highly processed foods, have lower intake of unprocessed foods, higher total energy consumption, and a greater likelihood of having an increased Body Mass Index (BMI) or being overweight or obese [26, 29, 30]. Furthermore, multinational fast-food production and consumption are experiencing a significant upsurge in Ghana through franchise models, leading to social differentiation through patterns of consumption, as well as the increased intake of highly processed foods in the rising urban middle class [31].

### Food consumption trends and drivers in Ghana

Recent research highlights the rapid increase in the total amount of food demanded in West Africa due to population growth and rising per capita income. Convenience foods, which are quick and easy to prepare and consume, are increasingly in demand across all income groups and countries in the region, particularly among urban populations, where limited time has been identified as a driver of consumption.[32]. In addition, while price remains a significant factor, consumers are also considering differentiated quality attributes, such as cleanliness, shelf life, cooking time, freshness, nutritional content, packaging, labelling, and presentation, as well as general concerns about the quality and health of food [32]. However, the studies show diverse developments and variations between the drivers for the consumption of multinational and local foods.

While previous studies have shown that Ghanaians value traditional food variety in their daily diets, consisting of local staples accompanied by seasonal vegetables and fruits prepared as soup or stew [15], processed and packaged foods are now ubiquitous in all households, including flavour cubes, canned tomatoes, imported rice, bread, canned or powdered milk, tea, Milo, sunflower oil, and canned fish or meat [15]. One reason identified for buying industrially processed foods was to bridge seasonal gaps in the availability of nutritious foods [15]. However, the increasing consumption of highly processed foods, such as bouillon cubes, when fresh food is unavailable poses challenges to the public health nutrition system and the local markets. It has been shown, that consumers primarily choose brands from multinational corporations based on taste, previous experiences, and expiry date, while perceptions of the nutritional quality of food are still largely based on traditional concepts of health and well-being. Food was described as "nutritious" if they give strength, energy, build the body, or build blood [15].

A cross-sectional survey conducted in Ghana revealed that despite having good general knowledge about traditional foods, consumers have limited knowledge regarding their nutritional composition [25]. Lower consumption of traditional foods was found to be potentially driven by convenience, economic status, and safety concerns associated with traditional foods. In contrast to multinationals' food products, consumers in Ghana are concerned about the generally unhygienic conditions under which food is prepared and sold in local markets, as this can lead to food-borne diseases such as cholera, typhoid, food poisoning, diarrhoea, avian influenza and swine fever [30]. Ghanaian consumers are concerned about the safety of food sold in local markets and fear it could harm their health. Consumers are most concerned about the use of pesticides in vegetables, artificial flavours and colours, bacterial contamination and harmful substances from plastic packaging [30, 31]. Demographic factors such as age and education did not significantly relate to specific attitudes, knowledge, and consumption towards local foods [25]. However, another study focused on consumer preferences of local chicken versus imported chicken found that the quality of the chicken in terms of freshness and taste, as well as ethnocentrism in favour of domestic production, can positively influence the consumption of local products [33].

Overall, there is limited research on the drivers behind the consumption of multinational corporation food products in Ghana. While some studies have focused on specific product categories, retail outlets or fast-food settings, there is a lack of sophisticated research that includes other factors that may influence the overall preference and desirability of these products.

## Methodology

### Study setting

The present study employed a maximum difference scaling experiment to investigate consumer preferences for multinational food corporations’ products over local foods in the Greater Accra Region of Ghana, which is the most populous and urbanized among the 16 administrative regions of Ghana. The region encompasses an area of approximately 3,245 square kilometres and has an estimated population of 5,446,237, with an urban population increase of about 37.7% between the years 2010 and 2021 [34]. Given that the Greater Accra Region serves as the political capital of Ghana, it is a major economic hub that heavily influences consumer behaviour in terms of multinational food corporations’ products[35].

### Study design and data collection

To conduct this study, we surveyed 200 consumers within the Greater Accra Region (Accra) over a period of three weeks in March/April 2023 using paper–pencil questionnaires. We utilized a random sampling technique to obtain the data collected through interviewer-administered questionnaire. This method aid the respondents in understanding the questions and writing out their responses. However, self-administration of the questionnaire was allowed upon request by some respondents to minimize potential interviewer bias. Respondents were approached at multinational supermarkets and international fast-food restaurants such as Shoprite Holdings Ltd, Barcelos Ghana, PICK ‘N PAY, Burger King, Massmart, Chicken Inn, SPAR, Kentucky Fried Chicken, Melcom Group, Pizza Hut, and Pizza Inn. Prior to data collection, permission to collect data was obtained in the form of written consent from the respondents after duly explaining to them the purpose of the study. Respondents were informed that their participation was voluntary, and they were at liberty to decide whether to participate or not in the study.

The survey questionnaire consisted of two sections. The first section, Section A, gathered socio-demographic characteristics of the respondents as well as reasons that inform consumers’ decision to select or choose products and/or meals from international food corporations and fast-food restaurant chains. The second section, Section B, focused on factors that influence consumers' choice of multinational food corporations' products over local foods. Prior to the main survey, a pilot study was conducted to identify possible challenges and problems during data collection, assess respondents' understanding of the various factors, manage the length of the questionnaire and respondents' reasons or basis for indicating their preferences. Adjustments were made to the questionnaire to address the respondents' issues and enhance comprehension while reducing information overload and cognitive burden [36].

The sample for this study consisted of 200 respondents who completed the survey. It is worth noting that the sample size exceeded the minimum sample size suggested by [36] for the number of choice scenarios presented in the questionnaire. According to their proposed sample size calculation, approximately 77 respondents were needed to accurately estimate preference weights. However, the obtained sample size was about two times larger than the minimum required. Therefore, all 200 respondents were included in the final analysis as there were no missing responses.

### Experimental design

Maximum difference scaling is a state-of-the-art approach for conducting consumer experiments [37]. Interest in using this method is growing in diverse areas [38] such as health [39–41], and environmental sustainability [42]. Researchers have discussed the potential for wider application of such experiments in food-related consumer research [43–45]. In this experimental design, each respondent is asked to select the most-preferred attribute and the least-preferred attribute from at least three profiles in a given choice set [46]. One of the main benefits of maximum difference scaling is its capacity to estimate the relative importance of all attributes on a common scale. Unlike traditional rating scale surveys, maximum difference scaling involves greater involvement and cognitive effort, which may help consumers focus when completing the choice task [47]. The appeal of maximum difference scaling relative to discrete choice experiments [48] has also been highlighted [43, 49].

To identify potential factors that could inform consumers' routine decision to choose multinational food corporations' products over local foods, an extensive literature review was conducted [15, 50–54], along with expert consultation involving food actors. Subsequently, a focus group discussion was conducted with 10 food actors and 30 potential consumers of multinational food corporations' and international fast-food restaurant products. The initial list of potential factors was narrowed down to 16 plausible attributes, which are presented in Table 1.

**Table 1:** Attributes considered in the maximum difference scaling experiment questionnaire

Attributes	Explanation
Nutrition content/ nutritional value	perceived ratio of carbohydrates, fats, proteins, and energy of a food
Image/ desirability	subjectively perceived image of a food as a representation of global lifestyles, foreignness, or non-localness, which is seen as prestigious and cosmopolitan
High in fibre and roughage	portion of plant foods, such as whole grains, nuts, seeds, legumes, fruits, and vegetables, present in a food
Taste/ flavour	expected or previously experienced taste of a food
Less preparation time of a meal	convenience of ready meals/take-away food that can be prepared in 5 to 15 min
Familiarity of a meal	frequency of consumption of a meal, with a threshold of at least once a week
Healthiness	subjectively perceived healthiness of a food
Social (family/friends eat)	socialization into, and cultural norms around, eating habits, including the diet consumed within the family or by friends
Food quality/packaging	subjective perception of the quality of the ingredients used in the food/meal. Multinational food is perceived as safer than conventional food (sold on the open market) and well packaged/covered
Availability	ready-to-eat meals are readily present at fast-food franchises and restaurants for purchase
Accessibility	convenience of not having to travel long distances to obtain the food
Affordability	consumer's ability to afford the cost of the food/meal
Convenience	convenience of ready-to-eat meals that can be bought in food franchises and restaurants close to where the consumer lives/works or can be ordered without walking (i.e., in a sedentary lifestyle)
Aroma/smell	favor sensation or reflection of the sense of taste associated with the food/meal
Texture	characteristics of a meal that can be felt with the fingers, tongue, palate, or teeth
Visual aesthetic	pleasing appearance of a meal

**Fig. 1:**
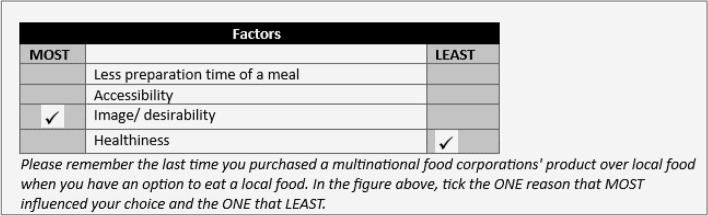
A sample completed maximum difference scaling experiment choice set as presented to respondents

To ensure manageable and comprehensible choice sets for the respondents, 20 choice sets were created using a balanced incomplete block design [55]. The balanced incomplete block design employed for $k$ attributes is denoted as $\left( {b,r,v,\lambda } \right)$ where $b$ is the number of choice sets (blocks), $r$ is the repetition per level, $v$ is the number of items in each choice set (block size) and $\lambda$ is the pair frequency. For example, the design noted as 20, 5, 4, 1 for 16 attributes has 20 choice sets, each attribute appears 5 times across all choice sets, each choice set contains four attributes, and each attribute appears once with each other. The 20 choice sets generated from the balanced incomplete block design contain four attributes per set. This approach mitigated the issue of cognitive overload and minimized the potential cognitive burden that may be induced by presenting too many attributes within each choice set [36, 56]. During the survey, each participant was presented with the 20 choice sets, with each set comprising four attributes, as depicted in Fig. 1. The respondents were required to express their preferences by selecting the "best" (most important) reason (attribute) and the "worst" (least important) attribute while considering purchasing a multinational food corporations’ product over local food (when there is a means or an option to eat local food) related to the situation described in Fig. 1. The situation was defined to standardize the reasons for considering purchasing a multinational food corporation's product over local food and to avoid confusion with special situations where people might think about directly comparing preferences for multinational food corporations' products to local foods as frequently encountered in discrete choice experiments [48], where respondents have to compare product descriptions and select one alternative in a choice set.

### Empirical strategy/ Data analysis

In a maximum difference scaling experiment, profiles are evaluated using a random utility framework [57, 58]. The choice frequencies for best and worst options in a choice set are used to compare the relative importance of different attributes. The Maximum difference model estimates the underlying utility of each choice.

To formalize this model, we denote $\hbar$ with $\left| \hbar \right| \ge 3$ as the finite set of potentially available options from a choice set and let $\psi \left( \hbar \right)$ denote the statistical experimental design, that is, the set of (sub)sets of choice options that occur in this study. For any set $Y \in \psi \left( \hbar \right),$ $Y \subseteq \hbar$ with $\left| Y \right| \ge 3$, let $P_{Y} \left( i \right)$ and $P_{Y} \left( j \right)$ denote the probability that respondents select a pair of items $i$ and $j$ from set $Y$, where $i$ is selected as the best and $j$ is selected as the worst, and the difference in utility between the two items is the maximum among all utility differences. Here $P_{Y} \left( {i,j} \right)$ is the probability that the item $i$ is selected as the best and item $j \ne i$ is selected as the worst.

By assuming that there is a scale ${\upmu }$ such that for all $i \in Y \in \psi \left( \hbar \right),$ where the value $\upmu \left( i \right)$ for an item $i$ is interpreted as the utility for that option, the best choice model can be formulated as

1.1 $$P_Y\left(i\right)=\frac{e^{\mathrm\mu\left(\mathrm i\right)}}{\sum_{Z\in Y}e^{\mathit\mu\mathit{\left(z\right)}}}$$

The parallel worst choice model can be reformulated as follows if we assume that there is a scale *v* such that for all $j \in Y \in \psi \left( \hbar \right),$

1.2 $$P_Y\left(j\right)=\frac{{e}^{v\left(j\right)}}{\sum_{Z\in Y}e^{v\left(z\right)}}$$

If both the corresponding choice probabilities on best and worst item satisfy all distinct pairs $i,j \in Y \in \psi \left( \hbar \right),$ then

$$P_{{\left\{ {i,j} \right\}}} \left( i \right){ = }P_{{\left\{ {i,j} \right\}}} \left( j \right),$$

and we obtain

1.3 $$P_Y\left(j\right)=\frac{e^{-\mathrm\mu\left(\mathrm i\right)}}{\sum_{Z\in Y}e^{-\mu\left(z\right)}}$$

Assume that the choice probabilities satisfy the corresponding best and worst model, and that the utility of a choice alternative in the selection of a best option is the negative of the utility of that option in the selection of a worst option, and this utility-scale $\upmu$ is such that for all $i,j \in Y \in \psi \left( \hbar \right),$ $i \ne j,$

1.4 $$P_{Y} \left( {i,j} \right) = \frac{{\mathop e\nolimits^{{\left[ {\upmu \left( i \right){ - \mu }\left( j \right)} \right]}} }}{{\sum\nolimits_{{\left\{ {p,q} \right\} \in Y}} {\mathop e\nolimits^{{\left[ {\upmu \left( p \right){ - \mu }\left( q \right)} \right]}} } }},$$

where $\upmu \left( i \right)$ is the systematic component of the utility of item $i,$ which is assumed to be $\upmu \left( i \right) = \beta_{i} X_{i}$, where $\beta_{i}$ is a preference coefficient to be estimated and $X_{i}$ is a dummy variable taking the value 1 if item $i$ is included in a choice set, and 0 otherwise. In this study, consumers independently select the attributes related to multinational food corporations’ products/meals they like and dislike the most when compared to local meals.

We fitted the maximum difference model to our data using JMP Pro Version 16.0. Statistical significance was measured at *p*-values of less than 0.001, 0.01, and 0.05. In the absence of *p*-values, statistical significance was measured at 95% confidence intervals (CIs) greater than or less than zero. A significant positive/negative preference coefficient indicates a high/low preference for a specific attribute. The sign of the preference coefficient indicates whether the plausible attribute has a positive or negative effect on utility. We compared the relative importance of the different attributes across attributes given the utility estimates (preference coefficients).

## Results

### Sample characteristics

Table 2 presents the demographic characteristics of the respondents as well as their frequency of consumption of products from multinational food corporations. The study comprised 200 participants with a median age of 26.5 years (interquartile range (IQR): 22–32 years). Most of the respondents were female (53%), unmarried (70.5%), childless (66.5%), and held a bachelor's degree (38%). These findings mirror the expected composition of the study population, as the Greater Accra region is known to have a distinct age distribution, with a higher proportion of young adults (aged 15–35 years) and a lower total fertility rate (2.2%) compared to other regions in Ghana [34]. Among the respondents, 38.5% reported frequently consuming products or meals from multinational food corporations or fast-food chains during the daytime, with 32.5% reporting occasional consumption, 16% reporting habitual consumption, 12.5% reporting infrequent consumption, and only 0.5% reporting never consuming such products. Respondents aged 15–29 years, females, unmarried individuals, childless individuals, and those with a bachelor's degree reported higher rates of frequent consumption, with 25.5%, 21%, 29.5%, 28%, and 16% of respondents in these respective categories reporting frequent consumption of multinational food corporation products or meals.

**Table 2:** Sample characteristics and frequency of consumption of products from international food corporations

Variable	Category	Respondents (*n* = 200)	Number of respondents by frequency of products/meals consumption				
			*Always*	*Often*	*Sometimes*	*Rarely*	*Never*
Age (years) median (IQR*)		26.5 (22–32)					
*Age (years)*	15 – 29	126 (63.0%)	18 (9.0%)	51 (25.5%)	44 (22.0%)	13 (6.5%)	0 (0.0%)
	30 – 49	67 (33.5%)	14 (7.0%)	23 (11.5%)	19 (9.5%)	10 (5.0%)	1 (0.5%)
	50 +	7 (3.5%)	0 (0.0%)	3 (1.5%)	2 (1.0%)	2 (1.0%)	0 (0.0%)
*Gender*	Male	93 (46.5%)	21 (10.5%)	34 (17.0%)	23 (11.5%)	14 (7.0%)	1 (0.5%)
	Female	106 (53.0%)	11 (5.5%)	42 (21.0%)	42 (21.0%)	11 (5.5%)	0 (0.0%)
	Diverse	1 (0.5%)	0 (0.0%)	1 (0.5%)	0 (0.0%)	0 (0.0%)	0 (0.0%)
*Educational level completed*	Primary or less	19 (9.5%)	6 (3.0%)	3 (1.5%)	5 (2.5%)	4 (2.0%)	1 (0.5%)
	Secondary School/SHS/SSS	57 (28.5%)	8 (4.0%)	22 (11.0%)	21 (10.5%)	6 (3.0%)	0 (0.0%)
	Diploma/HND	19 (9.5%)	3 (1.5%)	8 (4.0%)	7 (3.5%)	1 (0.5%)	0 (0.0%)
	Bachelors	76 (38.0%)	8 (4.0%)	32 (16.0%)	24 (12.0%)	12 (6.0%)	0 (0.0%)
	Masters	19 (9.5%)	6 (3.0%)	8 (4.0%)	5 (2.5%)	0 (0.0%)	0 (0.0%)
	Ph.D./DrPH	10 (5.0%)	1 (0.5%)	4 (2.0%)	3 (1.5%)	2 (1.0%)	0 (0.0%)
*Marital status*	Single	141 (70.5%)	25 (12.5%)	59 (29.5%)	44 (22.0%)	13 (6.5%)	0 (0.0%)
	Married	46 (23.0%)	5 (2.5%)	15 (7.5%)	17 (8.5%)	8 (4.0%)	1 (0.5%)
	Divorced	8 (4.0%)	2 (1.0%)	1 (0.5%)	4 (2.0%)	1 (0.5%)	0 (0.0%)
	Widowed	5 (2.5%)	0 (0.0%)	2 (1.0%)	0 (0.0%)	3 (1.5%)	0 (0.0%)
*Number of children*	None	133 (66.5%)	20 (10.0%)	56 (28.0%)	44 (22.0%)	13 (6.5%)	0 (0.0%)
	1 child	34 (17.0%)	5 (2.5%)	15 (7.5%)	11 (5.5%)	3 (1.5%)	0 (0.0%)
	2–3 children	27 (13.5%)	4 (2.0%)	6 (3.0%)	10 (5.0%)	6 (3.0%)	1 (0.5%)
	More than 3 children	6 (3.0%)	3 (1.5%)	0 (0.0%)	0 (0.0%)	3 (1.5%)	0 (0.0%)

^*^ *IQR* interquartile range

### Sample preference estimation

The results of the maximum difference model (Likelihood Ratio (LR) test statistic = 1203.665, *p* < 0.0001) demonstrated significant differences in preferences for the various attributes (Table 3). The estimated preference coefficients exhibited the expected sign within the 95% CIs. Each attribute was statistically significant (i.e., 95% CIs did not contain zero or were greater or less than zero); however, there was considerable overlap in the 95% CIs, indicating that certain attributes were not statistically different from each other. We interpret these results with respect to the model specification, beginning with the observation that the reference level was the attribute variable visual aesthetic. Preference coefficients for the attribute variables, such as aroma/smell, availability, food quality/packaging, healthiness, image/desirability, less preparation time of a meal, nutrition content/nutritional value, social (family/friends eat), taste/flavour, and texture, were mostly significant, indicating their influence on consumers' choice decisions. Specifically, the positive signs of the preference coefficients for attributes such as aroma/smell, food quality/packaging, healthiness, nutrition content/nutritional value, and taste/flavour indicated that consumers were more likely to choose products from multinational corporations over local foods. Conversely, negative signs for attributes such as availability, image/desirability, less preparation time of a meal, social (family/friends eat), and texture indicated disutility for choosing multinational corporations' products over local foods. This suggests that consumers tended to weigh the attributes of availability, image/desirability, less preparation time of a meal, social (family/friends eat), and texture against each other when selecting multinational food corporations' products/meals.

**Table 3:** Maximum difference model estimates of attributes that contribute to consumers choice of multinational corporations’ products over local foods

Attribute	Estimate	SE*	Lower 95%	Upper 95%
Accessibility	-0.0325	0.0442	-0.1192	0.0541
Affordability	0.0559	0.0439	-0.0300	0.1420
Aroma/smell	0.1089	0.0440	0.0226	0.1954
Availability	-0.1866	0.0440	-0.2730	-0.1003
Convenience	0.0060	0.0441	-0.0804	0.0925
Familiarity of a meal	-0.0616	0.0440	-0.1480	0.0245
Food quality/packaging	0.8053	0.0471	0.7134	0.8983
Healthiness	0.5995	0.0457	0.5102	0.6896
High in fibre and roughage	-0.0314	0.0436	-0.1169	0.0540
Image/desirability	-0.3284	0.0444	-0.4157	-0.2416
Less preparation time of a meal	-0.2328	0.0442	-0.3196	-0.1463
Nutrition content/ nutritional value	0.4076	0.0447	0.3201	0.4957
Social (family/friends eat)	-0.2734	0.0441	-0.3602	-0.1871
Taste/flavor	0.4918	0.0450	0.4037	0.5805
Texture	-0.8255	0.0470	-0.9184	-0.7337
**Model fits**				
L-R* test statistic	1203.66			
AIC*	18,258.42			
BIC*	18,352.37			
DF*	15			
*P-Value*	< 0.0001			
**Number of observations**	16,000			

^*^ *SE* standard error, *L-R* likelihood ratio, *AIC* Akaike information criteria, *BIC* Bayesian information criteria, *DF* degree of freedom

**Fig. 2:**
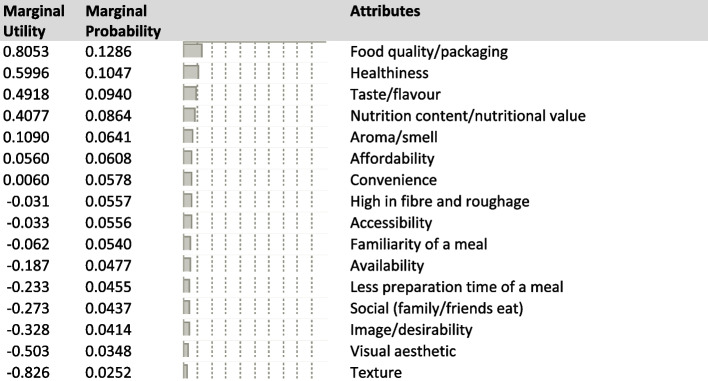
Marginal utility estimates and marginal probability of attributes that contribute to consumers choice of multinational corporations’ food products over local meals

To facilitate interpretation of the relative importance of each plausible attribute to consumers based on the magnitude of the preference coefficients, we have arranged the attributes in Fig. 2 in accordance with the marginal utility estimates (MUE) and their corresponding marginal probability (MP) values.

MUE represents the perceived importance of the matching level of the effect. Larger values imply that the level of the effect is of greater importance. MP represents the estimated probability that a consumer expresses a preference for the matching effect over all other effects. Our analysis revealed that food quality/packaging was the most important attribute (MUE: 0.8053; MP: 0.1286; 95% confidence interval [CI]: 0.7134, 0.8983), followed by healthiness (MUE: 0.5996; MP: 0.1047; 95% CI: 0.5102, 0.6896), taste/flavour (MUE: 0.4918; MP: 0.0940; 95% CI: 0.4037, 0.5805), nutrition content/nutritional value (MUE: 0.4077; MP: 0.0864; 95% CI: 0.3201, 0.4957), and aroma/smell (MUE: 0.1090; MP: 0.0641; 95% CI: 0.0226, 0.1954). However, we also observed negative relative importance for some attributes, such as availability (MUE: -0.187; MP: 0.0477; 95% CI: -0.2730, -0.1003), less preparation time of a meal (MUE: -0.233; MP: 0.0455; 95% CI: -0.3196, -0.1463), social (family/friends eat) (MUE: -0.273; MP: 0.0437; 95% CI: -0.3602, -0.1871), and image/desirability (MUE: -0.328; MP: 0.0414; 95% CI: -0.4157, -0.2416). Finally, texture was found to be the least important attribute (MUE: -0.826; MP: 0.0252; 95% CI: -0.9184, -0.7337).

Additionally, Table 4 provides a comparison of the greatest utility difference (GUD) among the preference weights for the attributes. GUD is defined as the maximum change in utility that can be achieved from an attribute, based on the plausible attributes included in the maximum difference experiment. Our results show that food quality/packaging had significantly the highest utility (GUD: 1.63088; *p* = 5e-113) compared to all other attributes related to consumers' preferences for multinational food corporations’ products/meals over local foods. Healthiness also had a significantly higher utility (GUD: 1.42512; *p* = 4.1e-91) but did not differ significantly from the taste/flavour attribute (*p* = 0.10109). The taste/flavour attribute had the next highest utility (GUD: 1.31736; *p* = 8.3e-80), followed by the nutrition content/nutritional value attribute (GUD: 1.23321; *p* = 3.4e-71), though it did not differ significantly from the taste/flavour attribute (*p* = 0.1957). The aroma/smell attribute had a lower but still significant utility (GUD: 0.93449; *p* = 1.7e-43), though it did not differ significantly from the convenience attribute (*p* = 0.10964).

**Table 4:** Preferences when two attributes that contribute to consumers choice of multinational food corporations’ products or meals are made available concurrently

Difference (Row-Column) Standard Error of Difference Wald * p*-Value	Accessibility	Affordability	Aroma/smell	Availability	Convenience	Familiarity of a meal	Food quality/packaging	Healthiness	High in fibre and roughage	Image/ desirability	Less preparation time of a meal	Nutrition content/ nutritional value	Social (family/friends eat)	Taste/ flavor	Texture	Visual aesthetic
Accessibility	0	-0.0885	-0.1415	0.15405	-0.0386	0.02912	-0.8379	-0.6321	-0.0011	0.29594	0.20026	-0.4402	0.24095	-0.5244	0.79298	0.47028
	0	0.06418	0.06475	0.06477	0.06454	0.06426	0.06665	0.06557	0.06398	0.06476	0.06467	0.06502	0.06438	0.06513	0.0666	0.06514
		0.16788	**0.02891**	**0.01744**	0.54991	0.65044	1.4e-35	9.2e-22	0.98615	5.03e-6	0.00197	1.5e-11	0.00018	1.1e-15	3.9e-32	6.2e-13
Affordability	0.08853	0	-0.053	0.24258	0.04994	0.11765	-0.7494	-0.5436	0.08742	0.38447	0.28879	-0.3517	0.32948	-0.4359	0.88151	0.55881
	0.06418	0	0.06414	0.06404	0.06422	0.06405	0.0667	0.06568	0.06374	0.06415	0.06427	0.06506	0.06423	0.06523	0.06667	0.06454
	0.16788		0.40882	0.00015	0.43684	0.06631	7.4e-29	1.7e-16	0.17032	2.23e-9	7.22e-6	6.84e-8	3.04e-7	2.7e-11	4.3e-39	6.8e-18
Aroma/smell	0.14151	0.05298	0	0.29557	0.10292	0.17064	-0.6964	-0.4906	0.1404	0.43746	0.34177	-0.2987	0.38246	-0.3829	0.93449	0.61179
	0.06475	0.06414	0	0.06431	0.06432	0.06437	0.06676	0.06538	0.06373	0.06452	0.06442	0.06468	0.06427	0.06523	0.06676	0.06503
	0.02891	0.40882		4.44e-6	0.10964	0.00806	3.7e-25	7.6e-14	0.02764	1.4e-11	1.19e-7	3.99e-6	2.9e-9	4.73e-9	1.7e-43	8.4e-21
Availability	-0.1541	-0.2426	-0.2956	0	-0.1926	-0.1249	-0.992	-0.7862	-0.1552	0.14189	0.04621	-0.5943	0.08689	-0.6784	0.63892	0.31622
	0.06477	0.06404	0.06431	0	0.06427	0.0641	0.06686	0.06577	0.06391	0.06439	0.06397	0.06507	0.06446	0.06529	0.06647	0.06474
	0.01744	0.00015	4.44e-6		0.00274	0.05135	1.7e-48	2.2e-32	0.01524	0.0276	0.47014	1e-19	0.17776	5.7e-25	1.2e-21	1.08e-6
Convenience	0.03859	-0.0499	-0.1029	0.19265	0	0.06772	-0.7993	-0.5936	0.03748	0.33454	0.23885	-0.4016	0.27954	-0.4858	0.83157	0.50887
	0.06454	0.06422	0.06432	0.06427	0	0.06448	0.06714	0.06624	0.06389	0.06455	0.06439	0.06477	0.06434	0.06504	0.06619	0.06494
	0.54991	0.43684	0.10964	0.00274		0.2937	3.9e-32	4.8e-19	0.55743	2.3e-7	0.00021	6.2e-10	1.43e-5	9.9e-14	1.6e-35	5.9e-15
Familiarity of a meal	-0.0291	-0.1177	-0.1706	0.12493	-0.0677	0	-0.867	-0.6613	-0.0302	0.26682	0.17114	-0.4694	0.21182	-0.5535	0.76385	0.44115
	0.06426	0.06405	0.06437	0.0641	0.06448	0	0.06657	0.06559	0.06384	0.06497	0.06452	0.06477	0.06444	0.0648	0.06669	0.0648
	0.65044	0.06631	0.00806	0.05135	0.2937		5.3e-38	1.3e-23	0.63583	0.00004	0.00802	5.1e-13	0.00102	1.8e-17	6.6e-30	1.1e-11
Food quality/packaging	0.8379	0.74937	0.69639	0.99196	0.79931	0.86702	0	0.20575	0.83679	1.13384	1.03816	0.39766	1.07885	0.31351	1.63088	1.30818
	0.06665	0.0667	0.06676	0.06686	0.06714	0.06657	0	0.06694	0.0666	0.06747	0.06704	0.06657	0.06707	0.06679	0.06989	0.06798
	1.4e-35	7.4e-29	3.7e-25	1.7e-48	3.9e-32	5.3e-38		0.00213	1.5e-35	2.7e-61	1.4e-52	2.52e-9	1.9e-56	2.77e-6	5e-113	5.5e-79
Healthiness	0.63215	0.54362	0.49064	0.7862	0.59356	0.66127	-0.2058	0	0.63104	0.92809	0.83241	0.19191	0.8731	0.10776	1.42512	1.10243
	0.06557	0.06568	0.06538	0.06577	0.06624	0.06559	0.06694	0	0.06544	0.06619	0.06589	0.06555	0.06599	0.06571	0.0686	0.06676
	9.2e-22	1.7e-16	7.6e-14	2.2e-32	4.8e-19	1.3e-23	0.00213		9.1e-22	1.2e-43	6.8e-36	0.00343	3.9e-39	0.10109	4.1e-91	2.6e-59
High in fibre and roughage	0.00111	-0.0874	-0.1404	0.15516	-0.0375	0.03023	-0.8368	-0.631	0	0.29705	0.20137	-0.4391	0.24206	-0.5233	0.79409	0.47139
	0.06398	0.06374	0.06373	0.06391	0.06389	0.06384	0.0666	0.06544	0	0.06419	0.06384	0.06475	0.06372	0.06491	0.06642	0.06468
	0.98615	0.17032	0.02764	0.01524	0.55743	0.63583	1.5e-35	9.1e-22		3.81e-6	0.00162	1.4e-11	0.00015	9.9e-16	2.2e-32	3.8e-13
Image/ desirability	-0.2959	-0.3845	-0.4375	-0.1419	-0.3345	-0.2668	-1.1338	-0.9281	-0.2971	0	-0.0957	-0.7362	-0.055	-0.8203	0.49703	0.17433
	0.06476	0.06415	0.06452	0.06439	0.06455	0.06497	0.06747	0.06619	0.06419	0	0.06447	0.06537	0.06458	0.06559	0.06639	0.06498
	5.03e-6	2.23e-9	1.4e-11	0.0276	2.3e-7	0.00004	2.7e-61	1.2e-43	3.81e-6		0.13785	5.6e-29	0.39453	3.1e-35	8.7e-14	0.00733
Less preparation time of a meal	-0.2003	-0.2888	-0.3418	-0.0462	-0.2389	-0.1711	-1.0382	-0.8324	-0.2014	0.09568	0	-0.6405	0.04069	-0.7246	0.59271	0.27002
	0.06467	0.06427	0.06442	0.06397	0.06439	0.06452	0.06704	0.06589	0.06384	0.06447	0	0.06519	0.06432	0.06543	0.06663	0.06512
	0.00197	7.22e-6	1.19e-7	0.47014	0.00021	0.00802	1.4e-52	6.8e-36	0.00162	0.13785		1.6e-22	0.52704	4.2e-28	8.6e-19	3.45e-5
Nutrition content/ nutritional value	0.44024	0.35171	0.29872	0.59429	0.40164	0.46936	-0.3977	-0.1919	0.43913	0.73618	0.6405	0	0.68118	-0.0841	1.23321	0.91051
	0.06502	0.06506	0.06468	0.06507	0.06477	0.06477	0.06657	0.06555	0.06475	0.06537	0.06519	0	0.06516	0.06502	0.06775	0.06587
	1.5e-11	6.84e-8	3.99e-6	1e-19	6.2e-10	5.1e-13	2.52e-9	0.00343	1.4e-11	5.6e-29	1.6e-22		3e-25	0.1957	3.4e-71	1.7e-42
Social (family/friends eat)	-0.2409	-0.3295	-0.3825	-0.0869	-0.2795	-0.2118	-1.0788	-0.8731	-0.2421	0.055	-0.0407	-0.6812	0	-0.7653	0.55203	0.22933
	0.06438	0.06423	0.06427	0.06446	0.06434	0.06444	0.06707	0.06599	0.06372	0.06458	0.06432	0.06516	0	0.06536	0.06639	0.06486
	0.00018	3.04e-7	2.9e-9	0.17776	1.43e-5	0.00102	1.9e-56	3.9e-39	0.00015	0.39453	0.52704	3e-25		3.7e-31	1.2e-16	0.00041
Taste/ flavor	0.52439	0.43586	0.38287	0.67844	0.48579	0.55351	-0.3135	-0.1078	0.52328	0.82033	0.72465	0.08415	0.76533	0	1.31736	0.99466
	0.06513	0.06523	0.06523	0.06529	0.06504	0.0648	0.06679	0.06571	0.06491	0.06559	0.06543	0.06502	0.06536	0	0.06808	0.06614
	1.1e-15	2.7e-11	4.73e-9	5.7e-25	9.9e-14	1.8e-17	2.77e-6	0.10109	9.9e-16	3.1e-35	4.2e-28	0.1957	3.7e-31		8.3e-80	9.1e-50
Texture	-0.793	-0.8815	-0.9345	-0.6389	-0.8316	-0.7639	-1.6309	-1.4251	-0.7941	-0.497	-0.5927	-1.2332	-0.552	-1.3174	0	-0.3227
	0.0666	0.06667	0.06676	0.06647	0.06619	0.06669	0.06989	0.0686	0.06642	0.06639	0.06663	0.06775	0.06639	0.06808	0	0.0667
	3.9e-32	4.3e-39	1.7e-43	1.2e-21	1.6e-35	6.6e-30	5e-113	4.1e-91	2.2e-32	8.7e-14	8.6e-19	3.4e-71	1.2e-16	8.3e-80		1.36e-6
Visual aesthetic	-0.4703	-0.5588	-0.6118	-0.3162	-0.5089	-0.4412	-1.3082	-1.1024	-0.4714	-0.1743	-0.27	-0.9105	-0.2293	-0.9947	0.3227	0
	0.06514	0.06454	0.06503	0.06474	0.06494	0.0648	0.06798	0.06676	0.06468	0.06498	0.06512	0.06587	0.06486	0.06614	0.0667	0
	6.2e-13	6.8e-18	8.4e-21	1.08e-6	5.9e-15	1.1e-11	5.5e-79	2.6e-59	3.8e-13	0.00733	3.45e-5	1.7e-42	0.00041	9.1e-50	1.36e-6	

In our study, we found that the affordability attribute had a positive utility value, indicating that it is a desirable attribute for consumers (GUD: 0.88151; *p* = 4.3e-39). However, this attribute did not significantly differ from other attributes such as high fibre and roughage, familiarity of a meal, convenience, and aroma/smell. When affordability was made available concurrently with other attributes, such as food quality/packaging, healthiness, nutrition content/nutritional value, and taste/flavour, consumers tended to trade it off (GUDs ranging from -0.3517 to -0.7494; ps < 0.05), suggesting that affordability may not be the deciding factor in their meal choices. Similarly, the convenience attribute had a significant positive utility value (GUD: 0.83257; *p* = 1.6e-35), but it did not differ significantly from the high fibre and roughage and familiarity of a meal attributes. When convenience was made available concurrently with other attributes such as food quality/packaging, healthiness, nutrition content/nutritional value, and taste/flavour, consumers tended to trade it off (GUDs ranging from -0.4016 to -0.7993; ps < 0.05). Overall, our findings suggest that consumers prioritize attributes such as food quality/packaging, healthiness, nutrition content/nutritional value, and taste/flavour over affordability and convenience when selecting international meal or food products over local options.

We also observed significant differences for the attribute variable high in fibre and roughage (DUE: 0.79409; *p* = 2.2e-32). However, when made available concurrently with the nutrition content/nutritional value attribute (GUD: -0.4391; *p* = 1.4e-11) and the taste/flavour attribute (GUD: -0.5233; *p* = 19.9e-16), it will be traded off. Similarly, the accessibility attribute was significantly different (GUD: 0.79298; *p* = 3.9e-32), but it will be traded off when made available concurrently with the aroma/smell attribute (GUD: -0.1415; *p* = 0.02891), healthiness (GUD: -0.6321; *p* = 9.2e-22), food quality/packaging (GUD: -0.8379; *p* = 1.4e-35), nutrition content/nutritional value (GUD: -0.4402; *p* = 1.5e-11) as well as taste/flavour (GUD: -0.5244; *p* = 31.1e-15). These findings suggest that the high in fibre and roughage attribute and the accessibility attribute are not as important as the other attributes when making food choices.

The study found that the attribute variable "familiarity of a meal" had a significant positive utility value (GUD: 0.76385; *p* = 6.6e-30), but it did not differ significantly from the attribute "high in fibre and roughage" (*p* = 0.63583) or "availability" (GUD: 0.63892; *p* = 1.2e-21), which did not differ significantly from the attribute "less preparation time of a meal" (*p* = 0.47014) or "social (family/friends eat)" (*p* = 0.17776). However, when made available concurrently with other attributes, familiarity of a meal was traded-off for food quality/packaging (GUD: -0.867; *p* = 5.3e-38), healthiness (GUD: -0.6613; *p* = 1.3e-23), nutrition content/nutritional value (GUD: -0.4694; *p* = 5.1e-13), and taste/flavour (GUD: -0.5535; *p* = 1.8e-17). Similarly, "less preparation time of a meal" had a significant utility value (GUD: 0.59271; *p* = 8.6e-19), but it did not differ significantly from the "social (family/friends eat)" attribute (*p* = 0.52704). However, when made available concurrently with other attributes, "less preparation time of a meal" was traded-off for nutrition content/nutritional value (GUD: -0.6405; *p* = 1.6e-22) and taste/flavour (GUD: -0.7246; *p* = 34.2e-28). Finally, the attribute variable "social (family/friends eat)" had a significant positive utility value (GUD: 0.55203; *p* = 91.2e-16), but it was traded-off for taste/flavour (GUD: -0.7653; *p* = 63.7e-31) when made available concurrently with this attribute.

The attribute variable "image/desirability" was found to have a significantly lower utility (GUD: 0.49703; *p* = 8.7e-14) than all other attributes related to multinational food corporations’ products/meals preference over local foods. However, this attribute did not differ significantly from the attributes of "less preparation time of a meal" (*p* = 0.13785) and "social (family/friends eat)" (*p* = 0.39453). These results suggest that although "image/desirability" can influence consumers' food choices, it is less valued than other attributes. It is noteworthy that the "image/desirability" attribute will be traded off when made available concurrently with "nutrition content/nutritional value" (GUD: -0.7362; *p* = 5.6e-29) and "taste/flavour" (GUD: -0.8203; *p* = 3.1e-35). Thus, consumers are likely to base their food choices on nutrition content/nutritional value and taste/flavour rather than the image/desirability of the meals/products.

## Discussion

The present study aimed to investigate the key factors that influence the food choices of urban Ghanaian consumers regarding multinational food corporations’ products over local foods. The findings revealed that food quality and safe packaging, perceived healthiness, taste and flavour, as well as nutritional value, were the main drivers of consumer preferences for multinational food products. Of these factors, food quality and safe packaging emerged as the criterion with the highest utility, suggesting that it is a significant aspect of consumer food choice decisions. This finding is notable because previous research has not identified this criterion as a primary driver of consumer preference. Previous studies have mostly focused on either the supermarket [26, 29] or local market context [25] and have not compared the preferences for multinational food products over local foods when there is an option to eat local foods. By posing this question, the study sheds light on the substitutive links between traditional and imported multinational foods and dishes, which are becoming increasingly salient in the Ghanaian food landscape. Studies investigating the preference for consuming local food have found that concerns about quality, safety, and health are important factors in consumers' decision to refrain from consuming locally produced and distributed food [25, 50]. This suggests that concerns about food safety, combined with the availability and affordability of industrially produced food, may lead consumers to view multinational corporations as offering safer substitutes to local food supplies. However, anxiety over contamination and foodborne illness underscores the need for reliable policies and food controls for both local and imported food products [50]. In addition, public authorities and public health nutritionists should invest in accompanying education campaigns on a broader understanding of quality, so that food safety does not become the sole criterion for consumer food choice decisions. Such campaigns could help prevent the consumption of multinational, highly processed foods from being seen as the default choice.

The results of the present study underscore the importance of perceived health and nutritional value as significant factors driving the preference of multinational food corporations' products over local foods among urban Ghanaian consumers. This observation aligns with previous research in other countries of the Global South that highlight how health claims, labelling, and marketing play a crucial role in shaping consumers' perceptions of the health value of industrially produced foods [32]. Moreover, traditional foods in Ghana are often endowed with cultural meanings related to health, such as building the body, energy, and blood [15], which further highlights the importance of perceived healthiness in food choices. However, while multinational corporations often aggressively market their products, promoting supposed knowledge about their health value, local foods are generally less well-known in terms of their nutritional values [25]. This knowledge gap suggests that there is a need to provide more nutrition education to consumers to make them aware of the benefits of consuming local healthy foods. Public health nutritionists and policymakers could help to promote the consumption of local foods by providing more nutrition education and increasing awareness about the nutritional values of traditional foods. This approach could encourage more conscious consumption choices and promote a shift towards healthier and more sustainable food choices in urban Ghanaian settings.

The findings of this study reveal that a significant proportion of the Ghanaian urban population studied frequently or occasionally consume food or dishes from multinational companies, with younger, educated, single, and childless females being the predominant consumers. These findings partly align with previous studies that have identified regular supermarket shoppers as being economically better off with secondary or tertiary education, but contrast with previous socio-economic characteristics such as married individuals and those living in larger households [28]. Our results therefore also suggests that lack of time to prepare fresh food is one of the main reasons for consuming food from MNCs in the city of Accra, as it is readily available, affordable, and convenient for this group [32]. It can be inferred that the population under study not only acquires highly processed food items, such as Maggi cubes, canned, or powdered milk, from outlets that distribute products of multinational corporations to compensate for seasonal gaps in locally available nutrient-rich food [15], but also increasingly consumes convenience food and ready-to-eat meals owing to time constraints. The escalating consumption of highly processed and pre-packaged foods in urbanized regions of emerging economies has been associated with adverse health and environmental impacts [15, 27] necessitating the involvement of government and other private sector stakeholders to address public health, nutrition, and sustainability issues. This obligation may involve implementing general awareness campaigns to promote healthy and sustainable diets, as well as targeted policy measures, such as incentives to incorporate more nutritious food items and meals into the product portfolio or developing effective waste management systems [12].

The previously prevalent assumption that multinational products are sought after for their symbolic value, which is based on their foreignness or non-localness, and perceived as prestigious and cosmopolitan [17, 18] cannot be fully supported by the results of this study. However, it should be noted that taste/flavour was also found to be a significant criterion for the consumption of food from multinational corporations. Taste is not only an individual experience and sensorially determined but also socially constructed [59]. Therefore, from an anthropological perspective on taste, it is plausible that taste, as an expression of socio-cultural change, may contribute to the preference for multinational food.

In conclusion, the present study suggests that consumers tend to favour multinational foods over local foods when food quality and safety, health and nutritional value, and taste attributes are addressed. It is recommended that targeted public health campaigns be implemented to raise awareness of the negative health impacts associated with the consumption of highly processed multinational foods. Additionally, education campaigns aimed at promoting informed and conscious food choices could contribute to a cultural shift towards a more comprehensive perception of food quality. These measures could potentially counteract the increasing trend of consuming highly processed foods in emerging countries. The findings and recommendations of this study may potentially be applicable to other urban contexts in sub-Saharan African countries where the dominance of multinational food products and outlets is similarly high, and urban food environments have undergone similar developments in recent decades [21]. Enlightening consumers and fostering a countermovement to corporate norm-setting regarding societal perceptions of nutritious and safe foods in these emerging markets are of paramount importance to promote conscious food consumption. Given the rapid growth and market power of multinational food corporations in countries of the Global South [9], this represents a potential point of intervention that can be addressed early on through national public health nutrition campaigns. Such campaigns can help proactively mitigate the negative health implications associated with high consumption of these products and contribute to addressing the effects of globalization processes in food supply on population health [22].

The present study has some limitations that should be acknowledged. Firstly, due to the design of the survey and the complexity of the questionnaire, the study did not undertake a detailed differentiation by product categories and outlets, such as supermarkets and fast-food chain restaurants. Therefore, the study cannot draw definitive conclusions about which products or outlets the respondents referred to in their answers. Future studies are required to conduct more differentiated analyses of the preferences of specific products against the background of their national or seasonal availability to provide a more detailed picture of consumer behaviour. Additionally, the study did not consider the availability of local substitutes for imported products, which may be a crucial factor influencing consumer preferences. Secondly, since preferences are heterogeneous among consumers, the study only identified one main consumer segment. Further research should aim to identify additional consumer segments and distinguish them based on their preferences for the identified attributes. In addition, a comparison between rural and urban consumer groups would be of interest to explore potential differences in the drivers of preference for local vs. multinational foods, considering socio-economic characteristics as well.

The study only investigates specific attributes and does not cover other important attributes such as price, shelf life, and freshness, among others. This may lead to biased utility estimates. Future studies should include these attributes to address excluded attribute biases. Though the results can potentially be applied to other regions due to the probability sampling technique employed, the geographical area covered is limited. Additionally, the study relied on consumer reports, and their integrity could not be verified. Further study should incorporate reveal preference data. Though self-administration of the questionnaire was allowed upon request, interview-administered survey may lead to potential interviewer bias. Though the maximum difference scaling is a theory-driven and a cutting-edge survey-based method for conducting consumer studies where there is a greater involvement and the amount of cognitive effort required may help consumers to focus when asked to complete a best–worst task, there may be an issue of force-choice set. Finally, the study was conducted exclusively in urban hotspots in Accra, which are close to business centres and universities. Therefore, the sample may be biased in terms of educational attainment, and further studies should collect data in other locations, including rural areas, to gain a broader understanding of consumer behaviour.

## Conclusions

In conclusion, the study provides important insights into the factors that drive consumer preferences for multinational and local foods in urban Accra. While the preference for multinational foods is mainly determined by the perceived quality and safety of packaging, healthiness, nutritional value, and taste, the findings also suggest that targeted measures could be taken to promote the consumption of local food. These could include establishing quality control systems in local production facilities and distribution outlets, promoting healthy eating campaigns, and encouraging multinational corporations to support waste management systems and offer more health-promoting product ranges. Additionally, consumer awareness campaigns could be implemented to raise awareness of the health risks associated with highly processed food, the nutritional values of local and industrial food, and a comprehensive understanding of food quality. By targeting the relevant attributes that determine preferences, these campaigns could successfully promote the consumption of local products and reduce the consumption of highly processed multinational products, ultimately improving public health, nutrition, and sustainability impacts in emerging economies.

## Data Availability

The datasets used and analysed during the current study are available from the corresponding author on reasonable request.

## Abbreviations

BMI: Body Mass Index
CIs: Confidence intervals
FDI: Foreign direct investment
GUD: Greatest utility difference
IQR: Interquartile range
LR: Likelihood ratio
MP: Marginal probability
MUE: Marginal utility estimates
NCDs: Con-communicable diseases

## References

[1] Hawkes C (2006). Uneven dietary development: linking the policies and processes of globalization with the nutrition transition, obesity and diet-related chronic diseases. Glob Health.

[2] Popkin BM, Adair LS, Ng SW (2012). Global nutrition transition and the pandemic of obesity in developing countries. Nutr Rev.

[3] Baum FE, Sanders DM, Fisher M, Anaf J, Freudenberg N, Friel S (2016). Assessing the health impact of transnational corporations: its importance and a framework. Glob Health.

[4] Bahn RA, Abebe GK (2020). Food retail expansion patterns in Sub-Saharan Africa and the Middle East and North Africa: Institutional and regional perspectives. Agribusiness.

[5] Wanyama R, Gödecke T, Chege CGK, Qaim M (2019). How important are supermarkets for the diets of the urban poor in Africa?. Food Sec.

[6] Hawkes C (2005). The role of foreign direct investment in the nutrition transition. Public Health Nutr.

[7] Baker P, Friel S (2016). Food systems transformations, ultra-processed food markets and the nutrition transition in Asia. Glob Health.

[8] Thow AM, Hawkes C (2009). The implications of trade liberalization for diet and health: a case study from Central America. Glob Health.

[9] Baker P, Machado P, Santos T, Sievert K, Backholer K, Hadjikakou M (2020). Ultra-processed foods and the nutrition transition: Global, regional and national trends, food systems transformations and political economy drivers. Obes Rev.

[10] Monteiro CA, Moubarac J-C, Cannon G, Ng SW, Popkin B (2013). Ultra-processed products are becoming dominant in the global food system. Obes Rev.

[11] Howse E, Hankey C, Bauman A, Freeman B (2021). Are young adults' discussions of public health nutrition policies associated with common food industry discourses? A qualitative pilot study. Austr New Zealand J Publ Health.

[12] Ahmed A, Lazo DPL, Alatinga KA, Gasparatos A. From Ampesie to French fries: systematising the characteristics, drivers and impacts of diet change in rapidly urbanising Accra. Sustain Sci. 2022:1–25. 10.1007/s11625-022-01195-y. 10.1007/s11625-022-01195-y PMC9379245 35990025

[13] Pulker CE, Trapp GS, Scott JA, Pollard CM (2019). The nature and quality of Australian supermarkets' policies that can impact public health nutrition, and evidence of their practical application: a cross-sectional study. Nutrients.

[14] Wills J (2023). Foundations for health promotion.

[15] Aberman N-L, Gelli A, Agandin J, Kufoalor D, Donovan J (2022). Putting consumers first in food systems analysis: identifying interventions to improve diets in rural Ghana. Food Sec.

[16] Popkin BM (2014). Nutrition, agriculture and the global food system in low and middle income countries. Food Policy.

[17] Hawkes C. Globalization, Food and Nutrition Transitions. WHO Commission on Social Determinants of Health. 2007. https://www.who.int/social_determinants/resources/gkn_hawkes.pdf?ua=1. Accessed 29 May 2021.

[18] Batra R, Ramaswamy V, Alden DL, Steenkamp J-BE, Ramachander S (2000). Effects of brand local and nonlocal origin on consumer attitudes in developing countries. J Consumer Psychol..

[19] Kennedy G, Nantel G, Shetty P. Globalization of food systems in developing countries: a synthesis of country case studies. 2004. 19178111

[20] Friel S (2021). Redressing the corporate cultivation of consumption: releasing the weapons of the structurally weak. Int J Health Policy Manage..

[21] HLPE. Nutrition and food systems: a report by the High Level Panel of Experts on Food Security and Nutrition of the Committee on World Food Security. 2017. http://www.fao.org/3/a-i7846e.pdf. Accessed 10 Mar 2023.

[22] Reardon T, Tschirley D, Liverpool-Tasie LSO, Awokuse T, Fanzo J, Minten B (2021). The processed food revolution in African food systems and the double burden of malnutrition. Glob Food Sec.

[23] Husmann C, Kubik Z. Foreign direct investment in the African food and agriculture sector: trends, determinants and impacts. 2019. https://bonndoc.ulb.uni-bonn.de/xmlui/bitstream/handle/20.500.11811/9628/ZEF_DP_274.pdf?sequence=1&isAllowed=y.

[24] Global Nutrition Report. Ghana. The burden of malnutrition at a glance. 2023. https://globalnutritionreport.org/resources/nutrition-profiles/africa/western-africa/ghana/. Accessed 10 Mar 2023.

[25] Laryea D, Akoto EY, Oduro I, Appaw WO (2016). Consumer perception of traditional foods in Ghana. Nutr Food Sci.

[26] Demmler KM, Ecker O, Qaim M (2018). Supermarket shopping and nutritional outcomes: a panel data analysis for urban Kenya. World Dev.

[27] Demmler KM, Qaim M. Africa's changing food environments and nutritional effects on adults and children. In: Biesalski HK, editor. Hidden hunger and the transformation of food systems: how to combat the double burden of malnutrition? 2020. p. 31–41. 10.1159/000507492. 10.1159/000507492 33502364

[28] Meng T, Florkowski WJ, Sarpong DB, Chinnan MS, Resurreccion AVA (2014). Consumer’s food shopping choice in Ghana: supermarket or traditional outlets?. Int Food Agribus Manage Assoc (IFAMA).

[29] Rischke R, Kimenju SC, Klasen S, Qaim M (2015). Supermarkets and food consumption patterns: The case of small towns in Kenya. Food Policy.

[30] Kimenju SC, Rischke R, Klasen S, Qaim M (2015). Do supermarkets contribute to the obesity pandemic in developing countries?. Public Health Nutr.

[31] Boafo J, Sarku R, Obodai J (2021). From the kitchen to fast food restaurants: the changing culture of food in urban Ghana. Food Stud: Interdiscip J..

[32] Staatz J, Hollinger F. West African food systems and changing consumer demands. Paris; 2016.

[33] Asante-Addo C, Weible D (2020). Imported versus domestic chicken consumption in Ghana: do attitudes and perceptions matter?. J Int Food Agribus Market.

[34] Ghana Statistical Service (GSS). Ghana 2021 Population and Housing Census: General Report. 2022. https://census2021.statsghana.gov.gh/gssmain/fileUpload/reportthemelist/Volume%203%20Highlights.pdf. Accessed 21 Mar 2023.

[35] Agyei-Mensah S, de Graft Aitkins A (2010). Epidemiological transition and the double burden of disease in Accra, Ghana. J Urban Health-Bull New York Acad Med..

[36] Hensher DA, Rose JM, Greene WH. Applied choice analysis: Cambridge University Press; 2015.

[37] Finn A, Louviere JJ (1992). Determining the appropriate response to evidence of public concern: the case of food safety. J Public Policy Mark.

[38] Louviere JJ, Flynn TN, Marley AAJ. Best-worst scaling: Cambridge University Press; 2015.

[39] Dobischok S, Metcalfe R, Matzinger E, Palis H, Marchand K, Harrison S (2023). Measuring the preferences of injectable opioid agonist treatment (iOAT) clients: Development of a person-centered scale (best-worst scaling). Int J Drug Policy.

[40] Xiong X, Dalziel K, Huang L, Rivero-Arias O (2023). Test-retest reliability of EQ-5D-Y-3L best-worst scaling choices of adolescents and adults. Value Health.

[41] Cheung KL, Wijnen BFM, Hollin IL, Janssen EM, Bridges JF, Evers SMAA, Hiligsmann M (2016). Using best-worst scaling to investigate preferences in health care. Pharmacoeconomics.

[42] van Schoubroeck S, Chacon L, Reynolds AM, Lavoine N, Hakovirta M, Gonzalez R (2023). Environmental sustainability perception toward obvious recovered waste content in paper-based packaging: An online and in-person survey best-worst scaling experiment. Resour Conserv Recycl.

[43] Aizaki H, Fogarty J (2023). R packages and tutorial for case 1 best–worst scaling. J Choice Modell.

[44] Rolfe J, Rajapaksa D, de Valck J, Star M (2023). Will greenhouse concerns impact meat consumption? Best-worst scaling analysis of Australian consumers. Food Qual Prefer.

[45] Jaeger SR, Jørgensen AS, Aaslyng MD, Bredie WL (2008). Best–worst scaling: An introduction and initial comparison with monadic rating for preference elicitation with food products. Food Qual Prefer.

[46] Najafzadeh M, Lynd LD, Davis JC, Bryan S, Anis A, Marra M, Marra CA (2012). Barriers to integrating personalized medicine into clinical practice: a best-worst scaling choice experiment. Genet Med.

[47] Cohen SA. Maximum Difference Scaling: Improved Measures of Importance and Preference for Segmentation. 2003. https://sawtoothsoftware.com/resources/technical-papers/maximum-difference-scaling-improved-measures-of-importance-and-preference-for--segmentation. Accessed 3 May 2023.

[48] Louviere JJ, Woodworth G (1983). Design and analysis of simulated consumer choice or allocation experiments: an approach based on aggregate data. J Mark Res.

[49] Cohen E (2009). Applying best-worst scaling to wine marketing. Int J Wine Bus Res.

[50] Omari R, Frempong G (2016). Food safety concerns of fast food consumers in urban Ghana. Appetite.

[51] Livingstone KM, Lamb KE, Abbott G, Worsley T, McNaughton SA (2020). Ranking of meal preferences and interactions with demographic characteristics: a discrete choice experiment in young adults. Int J Behavior Nutr Phys Act.

[52] Kamphuis CBM, de Bekker-Grob EW, van Lenthe FJ (2015). Factors affecting food choices of older adults from high and low socioeconomic groups: a discrete choice experiment. Am J Clin Nutr.

[53] Rusmevichientong P, Jaynes J, Chandler L (2021). Understanding influencing attributes of adolescent snack choices: Evidence from a discrete choice experiment. Food Qual Prefer.

[54] Powell PK, Durham J, Lawler S (2019). Food choices of young adults in the United States of America: a scoping review. Adv Nutr.

[55] Auger P, Devinney TM, Louviere JJ (2007). Using best-worst scaling methodology to investigate consumer ethical beliefs across countries. J Bus Ethics.

[56] Bech M, Kjaer T, Lauridsen J (2011). Does the number of choice sets matter? Results from a web survey applying a discrete choice experiment. Health Econ.

[57] McFadden D. Conditional logit analysis of qualitative choice behavior. Zarembka P. (Ed.), Frontiers in Econometrics, Academic Press. 1974. p. 105–42.

[58] Flynn TN, Marley A. Best-worst scaling: theory and methods. In: Hess S, Daly A, editors. Handbook of Choice Modelling: Edward Elgar Publishing; 2014. p. 178–201. 10.4337/9781781003152.00014.

[59] Højlund S (2015). Taste as a social sense: rethinking taste as a cultural activity. Flavour.

